# Predictive Virtual Infection Modeling of Fungal Immune Evasion in Human Whole Blood

**DOI:** 10.3389/fimmu.2018.00560

**Published:** 2018-03-21

**Authors:** Maria T. E. Prauße, Teresa Lehnert, Sandra Timme, Kerstin Hünniger, Ines Leonhardt, Oliver Kurzai, Marc Thilo Figge

**Affiliations:** ^1^Applied Systems Biology, Leibniz Institute for Natural Product Research and Infection Biology, Hans Knöll Institute (HKI), Jena, Germany; ^2^Faculty of Biological Sciences, Friedrich Schiller University Jena, Jena, Germany; ^3^Center for Sepsis Control and Care (CSCC), Jena University Hospital, Jena, Germany; ^4^Fungal Septomics, Leibniz Institute for Natural Product Research and Infection Biology, Hans Knöll Institute (HKI), Jena, Germany; ^5^Institute of Hygiene and Microbiology, University of Würzburg, Würzburg, Germany

**Keywords:** *Candida albicans*, *Candida glabrata*, immune evasion, state-based model, innate immune response, polymorphonuclear neutrophils, whole-blood infection assay

## Abstract

Bloodstream infections by the human-pathogenic fungi *Candida albicans* and *Candida glabrata* increasingly occur in hospitalized patients and are associated with high mortality rates. The early immune response against these fungi in human blood comprises a concerted action of humoral and cellular components of the innate immune system. Upon entering the blood, the majority of fungal cells will be eliminated by innate immune cells, i.e., neutrophils and monocytes. However, recent studies identified a population of fungal cells that can evade the immune response and thereby may disseminate and cause organ dissemination, which is frequently observed during candidemia. In this study, we investigate the so far unresolved mechanism of fungal immune evasion in human whole blood by testing hypotheses with the help of mathematical modeling. We use a previously established state-based virtual infection model for whole-blood infection with *C. albicans* to quantify the immune response and identified the fungal immune-evasion mechanism. While this process was assumed to be spontaneous in the previous model, we now hypothesize that the immune-evasion process is mediated by host factors and incorporate such a mechanism in the model. In particular, we propose, based on previous studies that the fungal immune-evasion mechanism could possibly arise through modification of the fungal surface by as of yet unknown proteins that are assumed to be secreted by activated neutrophils. To validate or reject any of the immune-evasion mechanisms, we compared the simulation of both immune-evasion models for different infection scenarios, i.e., infection of whole blood with either *C. albicans* or *C. glabrata* under non-neutropenic and neutropenic conditions. We found that under non-neutropenic conditions, both immune-evasion models fit the experimental data from whole-blood infection with *C. albicans* and *C. glabrata*. However, differences between the immune-evasion models could be observed for the infection outcome under neutropenic conditions with respect to the distribution of fungal cells across the immune cells. Based on these predictions, we suggested specific experimental studies that might allow for the validation or rejection of the proposed immune-evasion mechanism.

## Introduction

Even though pathogenic microbes constantly colonize the human skin or are inhaled, the human immune system is usually able to protect the body against infections. Thus, immunocompromised individuals have an increased risk for infections by opportunistic pathogens (1). In case of injuries or disturbed cellular integrity, the pathogens can easily overcome physical skin barriers and/or mucosal surfaces, and enter the host tissue or the blood stream (2, 3). Innate immune responses defend the host against microbial invaders (4–6), however, the exact interplay between pathogens and the immune defense is in many cases not fully resolved (7, 8). In order to investigate such unknown mechanisms, mathematical modeling is an appropriate approach to investigate complex biological systems at a quantitative level. Furthermore, mathematical models allow for hypothesis testing by varying single parameters or comparing various possible scenarios. This approach allows going beyond experimental limitations, for example, by quantifying biological processes that are not amenable to a direct measurement in experiment. Moreover, ethical concerns and financial efforts of experimental studies can be considerably reduced by computer simulations, because systematic variations of model parameters allow narrowing down the number and kind of further experimental investigations necessary to identify causal relationships responsible for experimentally observed effects (9). The iterative cycle of such a systems biology approach combines wet-lab and dry-lab experiments to their best advantage (10, 11).

In previous studies, we have applied a systems biology approach to investigate the complex interaction of the human-pathogenic fungus, *Candida albicans* with innate immune cells in human whole blood (12, 13). Interestingly, we observed that a relatively high proportion of *C. albicans* can survive in human blood and evades the immune response by a so far unknown mechanism. The experimental part of this study comprised human whole-blood infection assays, where blood samples from healthy donors were infected with fungal cells to acquire time-resolved data on the interaction of *C. albicans* with immune cells as well as fungal survival over the course of infection. Based on these experimental results, a bio-mathematical model was developed using a state-based modeling approach (12, 13). The model is composed of states that represent different *C. albicans* cell populations of the biological system. These include alive and killed *C. albicans* cells, which are either in extracellular space or phagocytosed by the immune cells, i.e., PMN or monocytes. Moreover, the model represents a population of fungal cells that can evade the immune defense, since these cells appear to be neither phagocytosed by immune cells nor killed extracellularly. Transitions between various states of cell populations can occur and these state changes represent biological processes like phagocytosis and killing. In the original state-based model (SBM), transition rates were defined to characterize the different transitions between the states, which represent the biological processes. The *a priori* unknown values for these transition rates were evaluated by applying the global parameter estimation algorithm *Simulated Annealing* that is based on the *Metropolis Monte Carlo* scheme (12, 13). This algorithm explores the space of transition rates and searches for the global minimum of the fitting error, i.e., the deviation between the simulated and experimentally measured kinetics, and by that yields values for the transition rates that together achieve optimal agreement between these kinetics. The resulting rates indicated that the larger number of *C. albicans* cells inside PMN, in comparison to the much smaller number of fungal cells inside monocytes, is not merely a consequence of the higher number of PMN than monocytes, but is also due to a larger phagocytosis rate of PMN compared to monocytes. This quantification, which is not directly accessible from the experimental data alone, allowed us to generally conclude that elimination of *C. albicans* cells in human blood is governed by PMN.

**Figure 1:**
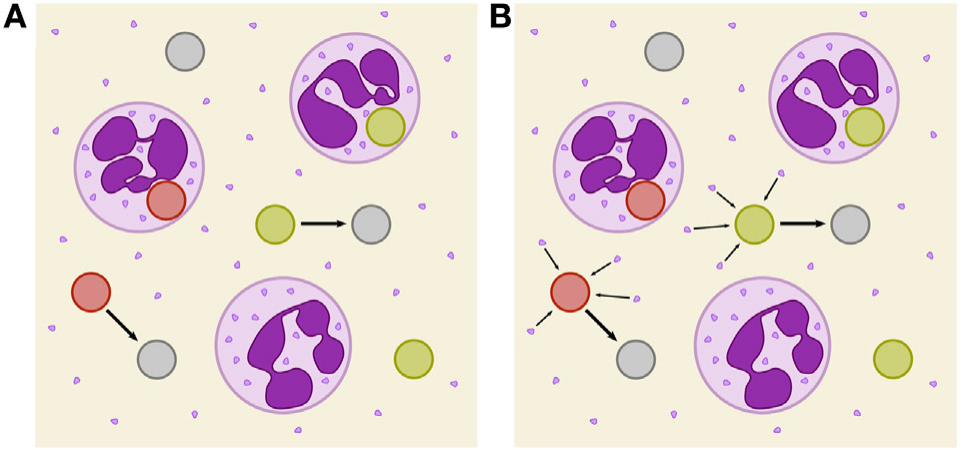
Schematic depiction of two immune-evasion mechanisms. PMN (purple) with granula and fungal cells are either alive (green), killed (red), or immune evasive (gray). **(A)** Illustration of spontaneously evading fungal cells. **(B)** Illustration of the PMN-mediated immune-evasion mechanism, which is associated with degranulation on first-time phagocytosis of fungal cells by PMN. Degranulation is assumed to mediate the release of proteins into extracellular space that enables fungal cells to evade subsequent killing and phagocytosis by modification of their surface.

In the SBM, fungal cells that evaded the immune response were assumed to undergo a spontaneous process with a constant transition rate and we will refer to it as *spon-IE model* from now on (see Figure 1A). While the exact mechanism causing immune evasion of *C. albicans* in human blood has not been identified yet, our previous studies already allowed for the rejection of various hypotheses. In the work by Hünniger et al. (12) it has been shown that the non-filamentous *efg1*Δ, *cph1*Δ mutant of *C. albicans*, and even thimerosal-killed *C. albicans* yeast cells are both able to evade the immune response. These observations imply that the fungal cells do not play an active role in the acquisition of immune-evasive properties. Therefore, we addressed aspects of the host. However, we found that the addition of fresh blood of the same donor to an infected blood sample after 2 h did not result in higher elimination of fungal cells, implying that the hypothesis of early PMN exhaustion in the infection assay could be rejected. Additionally, we observed that during the 4 h of whole-blood infection the number of immune cells remained fairly constant. Thus, acquisition of immune evasion by fungal cells inside the phagocytes, which might then be followed by the destruction of phagocytic immune cells, appears to be unlikely. This lytic escape mechanism, which has been observed for macrophages (14), has not been reported for human PMN in *C. albicans* infection.

In this study, we investigate the unresolved mechanism of immune evasion by pathogens in human whole blood. This is realized by making predictions based on mathematical modeling of the infection kinetics and by comparing various infection scenarios that may be tested in experiment. Based on our previously developed state-based virtual infection model (12, 13), we hypothesize that the immune-evasion process is mediated by host factors and incorporate such a mechanism in the model. Our hypothesis is motivated by the experimental observation that even thimerosal-killed *C. albicans* cells can acquire immune-evasive properties. Thus, pathogen immune evasion may be actively driven by the host. Although PMN are the main actors in the defense against *C. albicans*, immune cells also have been shown to cause remodeling of the *C. albicans* cell wall (15). However, while it is known that PMN degranulation is associated with the release of antimicrobial effector proteins that can kill *C. albicans* cells in extracellular space (16, 17), the consequences of the cell wall remodeling is yet not clear, e.g., whether or not it enables the immune evasion by the pathogen. We here consider the possibility that PMN degranulation is associated with the secretion of effector molecules that may cause immune evasion. We investigate the possibility that these PMN-derived molecules may change the pathogen surface and thereby render the pathogen undetectable for immune cells (see Figure 1B). We will refer to the model that assumes a PMN-mediated evasion mechanism as *PMNmed-IE model* in the following.

The PMNmed-IE model will be compared with the spon-IE model by simulating the immune response to pathogens in healthy individuals as well as in virtual patients with neutropenia. Furthermore, we also extend this analysis to the fungus *C. glabrata*, which attributes to the rise of microbial infection in the clinics, especially in elderly individuals and immunocompromised patients (18). The two fungal pathogens are part of the normal microbial flora of the majority of people and remain in a commensal state under healthy conditions (19). *C. albicans* and *C. glabrata*, respectively, rank first and second in isolation frequency in humans (20) and in immunocompromised patients can switch into a pathogenic state, overcome physical barriers, enter the bloodstream, and disseminate throughout the body (4, 7). In blood, the microorganisms are attacked and cleared by the innate immune response. However, we find that both pathogens—albeit to a different quantitative extent—have the ability to evade the immune response. This emphasizes once more the importance of investigating immune-evasion mechanisms by mathematical modeling in order to generate testable hypothesis that may be checked in experiment and ultimately enable medical intervention that cuts the pathogen escape route in and subsequent dissemination from human whole blood.

## Materials and Methods

### Ethics Statement

This study was conducted according to the principles expressed in the Declaration of Helsinki. All protocols were approved by the Ethics Committee of the University Hospital Jena (permit number: 273-12/09). Written informed consent was obtained from all blood donors.

### Fungal Strains and Culture

The GFP expressing *C. albicans* strain was constructed as described in Hünniger et al. (12) and grown in liquid yeast extract-peptone-dextrose (YPD) medium at 30°C. The GFP expressing *C. glabrata* strain (21) was incubated at 37°C in YPD medium. After overnight culture both strains were reseeded in fresh YPD medium followed by growing at 30 and 37°C, respectively, until they reached the mid-log-phase. Finally, the fungal cells were washed and harvested in HBSS until use.

### Human Whole Blood Infection Assay

Human peripheral blood samples from healthy individuals were infected with either *C. albicans* or *C. glabrata*. The assay was performed as described previously (12). In short, 1⋅10^6^ *Candida* cells were added per ml of anti-coagulated blood and incubated at 37°C with gentle rotation for indicated time points. Subsequent to the confrontation, samples were maintained at 4°C and further analyzed by flow cytometry. Flow cytometry gating strategy was performed as previously described using FlowJo 7.6.4 software to investigate the distribution of fungal cells in human blood (12). Survival of fungal cells was determined in a plating assay by analysis of recovered colony forming units after plating appropriate dilutions of all time points on YPD agar plates.

### SBM of Whole-Blood Infection

Recently, we established a virtual infection model to simulate the immune response against the fungal pathogen *C. albicans* in human whole blood (12, 13). This enabled us to quantify innate effector mechanisms as well as *C. albicans* immune evasion based on experimental data as obtained by FACS analysis and survival assays during a time course of 4 h. The time-resolved data comprised *C. albicans* viability as well as its association to innate immune cells, i.e., monocytes and PMN. In the SBM, immune cells and fungal cells can populate specific states. We identified five combined units of these states that could be directly compared with the experimentally measured cell populations. The combined unit *P_E_* involves all extracellular pathogens and is given by

(1) $$P_{E}\equiv P_{AE}+P_{KE}+P_{AIE}+P_{KIE}$$

Here, the states *P_AE_* and *P_KE_* represent extracellular cells that are alive and killed, respectively. The states *P_AIE_* and *P_KIE_* describe pathogens that are either alive and evade the immune response or kill and evade the immune response. Note that alive extracellular cells do not comprise alive immune-evasive cells and that these combined units are excluding each other.

Pathogens *P_AE_* and *P_KE_* can be phagocytozed by immune cells and in the SBM we account for phagocytosis by monocytes (M) and PMN (N), where the latter may also be referred to as neutrophils and are, therefore, labeled with N. An intracellular pathogen is either phagocytosed by a PMN

(2) $$P_{N}\equiv \sum_{i\geq 0}\sum_{j\geq 0}(i+j)N_{i,j},$$

or by a monocyte

(3) $$P_{M}\equiv \sum_{i\geq 0}\sum_{j\geq 0}(i+j)M_{i,j}.$$

Here, the indices *i* and *j* refer to the immune cell state that is defined by the number of internalized alive and killed pathogens, respectively. The combined unit of killed pathogens is given by

(4) $$P_{K}\equiv P_{KE}+P_{KIE}+\sum_{i\geq 0}\sum_{j\geq 0}(M_{i,j}+N_{i,j})j\;,$$

whereas the combined unit of alive pathogens is defined by

(5) $$P_{A}\equiv P_{AE}+P_{AIE}+\sum_{i\geq 0}\sum_{j\geq 0}(M_{i,j}+N_{i,j})i\;.$$

Note that the total number of pathogens is given by *P* ≡ *P_E_ + P_N_ + P_M_ + P_KIE_* or *P* ≡ *P_K_ + P_A_*.

The states are connected by transitions that indicate possible state changes and thereby enable to simulate the dynamics of the model (see Figure S1 in Supplementary Material). Transition rates characterize these state changes and are defined as the probability of a transition per simulation time step Δ*t*. The SBM by Hünniger et al. (12) and Lehnert et al. (13) distinguished a rate for first and subsequent phagocytosis events by PMN, since it was assumed that a phagocytosis event activates the PMN and leads to a higher phagocytosis rate. Since this fact is not experimentally validated for whole-blood infection with *C. glabrata*, we here implement a single phagocytosis rate of PMN that accounts for both, first and subsequent phagocytosis events. Therefore, the SBM of whole-blood infection comprises seven different transition rates that are given by the phagocytosis rate ϕ*_M_* of monocytes, the phagocytosis rate ϕ*_N_* of PMN, the intracellular killing rates κ*_M_* and κ*_N_* of both monocytes and PMN, the transition rates γ and ${\bar{\kappa }}_{EK}$, which define the extracellular killing, and the spontaneous immune-evasion rate ρ (see Table S1 in Supplementary Material). As already noted in our previous study (12), occasional filamentation of fungal cells but no budding could be observed in samples of blood smears. Therefore, proliferation of fungal cells is not included in the SBM. An overview of the SBM simulation algorithm is briefly described in Section S1 in Supplementary Material and schematically illustrated in Figure S1 in Supplementary Material. For a detailed description of the SBM, including the definition of rates for state transitions and their estimation by the *Simulated Annealing* algorithm that is based on the *Metropolis Monte Carlo* scheme (22, 23), we refer to our previous studies by Hünniger et al. (12) and Lehnert et al. (13). Here, we briefly mention that the values of the transition rates in the virtual infection model were estimated such that deviations from the kinetics of the combined units as obtained from the experiments are minimized. A brief overview of the parameter estimation algorithm is given in Section S2 and Figure S2 in Supplementary Material.

Our object-oriented framework combining the SBM simulation algorithm and the parameter estimation is implemented in the programming language C++ and available for download from https://asbdata.hki-jena.de/publidata/PrausseEtAl2018_FrontImmunol/.

### Modeling of Immune Evasion by Pathogens

As was observed in our previous analysis for *C. albicans*, pathogens can evade the immune response in the states alive (*P_AIE_*) or killed (*P_KIE_*), i.e., these cells can neither be phagocytosed nor killed by PMN and monocytes, and their total number is denoted by *P_IE_* ≡*P_KIE_* + *P_AIE_* (12). Note that immune evasion of *C. albicans* in human whole blood was first predicted by our state-based virtual infection model and then also verified experimentally. Since the mechanisms of the immune evasion could not be identified yet, this process was assumed to occur spontaneously with time-independent transition rate

(6) ρ=constant

and we refer to this model as spon-IE model. In this study, spontaneous immune evasion of pathogens (see Figure 1A) was compared to an immune-evasion mechanism, which was assumed to be mediated by PMN. Since PMN secrete antimicrobial peptides upon initial phagocytosis of pathogens, we speculated that these pathogens may also secrete proteins that can mediate the immune evasion (see Figure 1B), e.g., inducing alterations of pathogens by modulating its molecular surface. We accounted for this mechanism in the SBM by replacing the constant transition rate of the spon-IE model with the time-dependent rate

(7) $$\rho (t=n\Delta t)=\bar{\rho }\sum_{m=0}^{n}\frac{N_{NP}(t^{\prime }=m\Delta t)}{G_{\left(0,0\right)}(0)}\cdot \text{exp}(-\gamma_{R}\cdot \Delta t(n-m))$$

in the PMNmed-IE model. In close analogy to the rate of extracellular killing of pathogens by antimicrobial peptide-release from PMN (1), Eq. 7 represents the rate of pathogen immune evasion at time *t* as mediated by the sum of PMN-released proteins upon first phagocytosis events (*N_NP_*) up to time point *t*. Note that the simulation algorithm performs *n* simulation steps with step size Δ*t* to calculate the system dynamics at time point *t* = *n*Δ*t*. The impact of secreted molecules is determined by the parameters $\bar{\rho }$ and γ*_R_*, where the latter describes the half-life associated with the molecular degradation, such that the molecules’ immune-evasive effect is exponentially decreasing after their release at time *t'* = *m*Δ*t*. Therefore, the PMNmed-IE model comprises eight parameters, i.e., one more rate than the spon-IE model for spontaneous immune-evasion processes.

### Simulation of Virtual Patients With Neutropenia

In order to study the difference between the two models, spon-IE and PMNmed-IE, we simulated infection scenarios in human whole blood under neutropenic conditions. More specifically, virtual patients were considered with gradually decreasing amounts of PMN within the range of medically established severity levels of neutropenia (24) (see Table 1) and the impact of these conditions was compared with regards to the two mechanisms of immune evasion. The simulation algorithm described in Lehnert et al. (13) was applied to human whole-blood samples of 1 ml containing 5 ⋅10^5^ monocytes and 1 ⋅10^6^ pathogens. For each infection scenario, we performed 50 simulations with transition rate values that were randomly sampled within their respective SD.

**Table 1:** Number of PMN per ml blood for different severity levels of neutropenia.

State of disease	PMN (1/ml)
Healthy	1.8 ⋅10^6^–8 ⋅10^6^
Mild neutropenia	<1.5 ⋅10^6^
Moderate neutropenia	<1 ⋅10^6^
Severe neutropenia	<5 ⋅10^5^

## Results

### Whole-Blood Infection Show Pathogen-Specific Immune Response Kinetics

**Figure 2:**
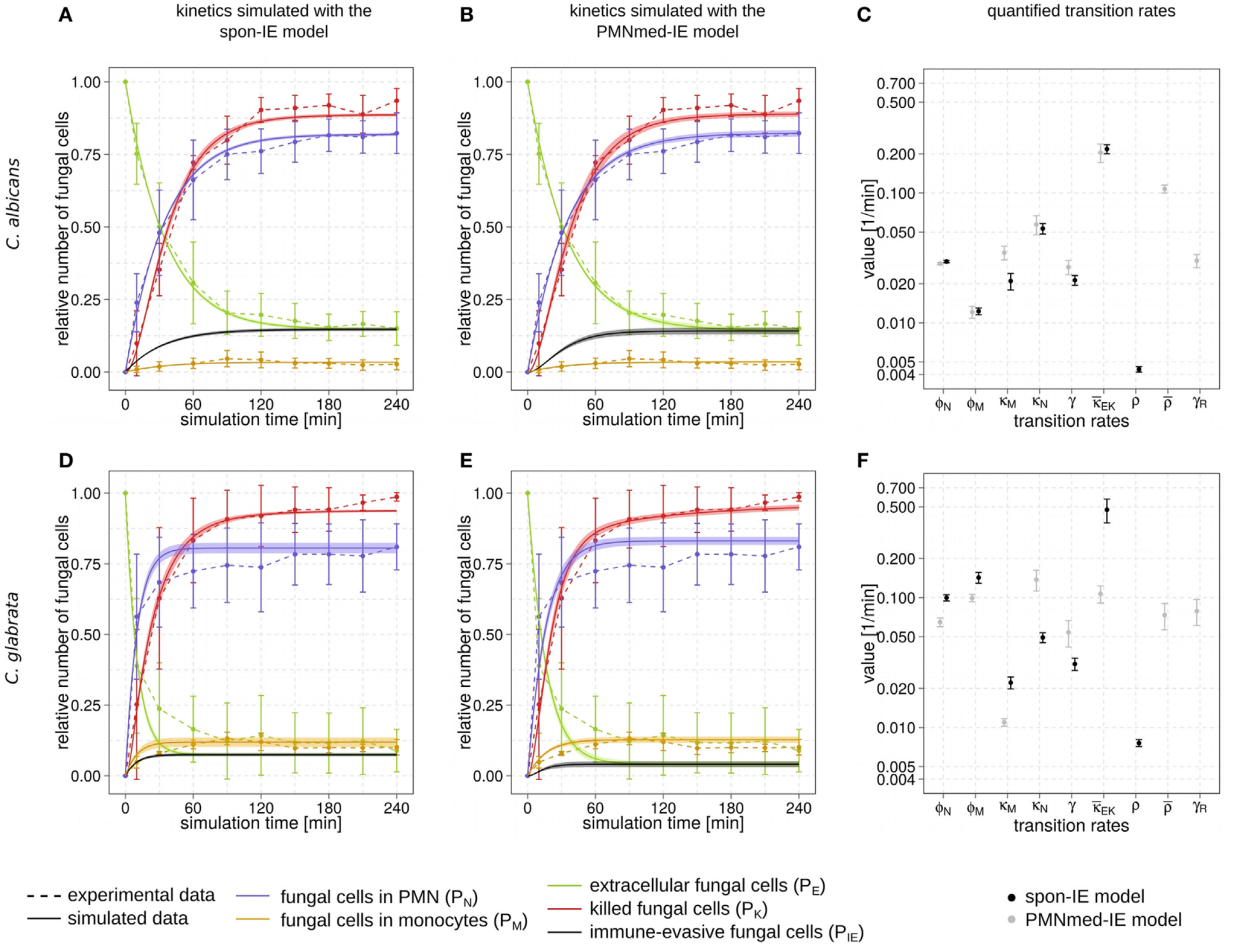
Kinetics of combined units and corresponding transition rate values of the spon-IE model and the PMNmed-IE model. Experimental data from whole-blood infection assays (dashed lines) with corresponding SDs are compared to the simulated data (solid lines) by the spon-IE model (left column) and the PMNmed-IE model (middle column). The thickness of solid lines indicates the mean ± SD of 50 simulations with transition rate values that were randomly sampled within their corresponding SD. Mean values (data points) and SDs (error bars) of transition rates (right column) were quantified by the global parameter estimation algorithm *Simulated Annealing* based on *Metropolis Monte Carlo* scheme for the spon-IE model (black data points) and PMNmed-IE model (gray data points). The transition rates are given by the phagocytosis rate ϕ*_N_* of PMN and the phagocytosis rate ϕ*_M_* of monocytes, the intracellular killing rates κ*_M_* and κ*_N_* of both monocytes and PMN, the transition rates γ and ${\bar{\kappa }}_{EK}$ which define the extracellular killing, and the spontaneous immune-evasion rate ρ and the PMN-mediated immune-evasion rates $\bar{\rho }$ and γ*_R_*, respectively. The time-course of the relative number of killed pathogens (*P_K_*), which are indicated by red dashed lines, were experimentally measured by survival assays. The relative number of fungal cells that were associated with monocytes (*P_M_*), PMN (*P_N_*), or in extracellular space (*P_E_*) were measured by association assays and indicated by orange, blue, or green dashed lines, respectively. The experimental results were compared with the corresponding combined units calculated for the simulated data. Black solid lines refer to the simulated time-course of immune-evasive fungal cells (*P_IE_*). Kinetics of a *Candida albicans* infection simulated by **(A)** the spon-IE model and **(B)** the PMNmed-IE model. **(C)** Transition rates quantified by both models for a *C. albicans* infection. Kinetics of a *Candida glabrata* infection simulated by **(D)** the spon-IE model and **(E)** the PMNmed-IE model. **(F)** Transition rates quantified by both models for a *C. glabrata* infection.

Whole-blood infection assays were performed for the two fungal pathogens, *C. albicans* and *C. glabrata*. At specific time points, whole-blood samples were analyzed using flow cytometry and survival assays to acquire time-resolved data for the association between pathogens and immune cells as well as viability of the pathogens. Figures 2A,B,D,E depict these experimental data (dashed lines) for *C. albicans* and *C. glabrata*, respectively.

Comparing the two pathogens, the fraction of extracellular fungal cells at 4 h post infection was highest for *C. albicans* with 15 ± 5.8%and lowest for *C. glabrata* with 8.9 ± 7.5%, where the sub-populations of alive and killed cells are comparable in size (see Figures 2A,B,D,E). In the case of an infection with *C. albicans*, a fraction of 6.5 ± 4.2% cells still remained alive at 4 h post infection, whereas survival assays revealed that 1.3 ± 1.5% of *C. glabrata* cells were not killed at that time point. Interestingly, the association of fungal cells to monocytes was markedly higher for *C. glabrata* with a fraction of 10.1 ± 2.7% compared to *C. albicans* with a fraction of 2.7 ± 1.9%. Furthermore, *C. albicans* showed only a slightly higher association of 82.3 ± 7.0% to PMN than *C. glabrata* (81.0 ± 8.1%), as was previously observed by Duggan et al. (25). Nevertheless, for both pathogens, the fraction of association to PMN was dominant over association to monocytes, i.e., by a factor eight for *C. glabrata* and by a factor 30 for *C. albicans*. Furthermore, Hopke et al. showed that degranulation of PMN has an impact on cell wall modulation in fungi, but whether this could enable pathogenic immune evasion is still unclear (15). These findings motivated our decision to focus on a PMN-mediated immune-evasion mechanism in comparison to spontaneous immune evasion.

### Spontaneous and PMN-Mediated Immune Evasion in Agreement With Experimental Data

We investigated the possibility that PMN secrete upon initial phagocytosis of pathogen proteins that can mediate immune evasion, e.g., inducing alterations of the surface of pathogens (15) (see Figure 1B). This mechanism was studied by applying mathematical modeling for hypothesis testing, i.e., we compared the impact of spontaneous versus PMN-mediated immune evasion on the infection outcome. To this end, we modified a previously implemented state-based virtual infection model (12, 13) to realize the PMN-mediated evasion mechanism. We refer to this model as *PMNmed-IE model* to distinguish it from the previously modeled spontaneous immune evasion, which we refer to as *spon-IE model*.

The transition rate values of the SBM were determined by the global parameter estimation algorithm *Simulated Annealing* based on *Metropolis Monte Carlo* scheme. This algorithm aims at searching for the optimal agreement between the simulated kinetics and the experimental data obtained from the whole-blood infection assays. The resulting transition rate values of both models are given in the Tables S2 and S3 in Supplementary Material and the corresponding simulated kinetics are depicted in Figure 2. Here, the experimental kinetics correspond to the combined units introduced in the Section “Materials and Methods” plotted in Figure 2. The excellent agreement between experiment and simulation can be seen for the whole-blood infection assays with either *C. albicans* (see Figures 2A,B) or *C. glabrata* (see Figures 2D,E) with their transition rate values in Figures 2C,F.

**Figure 3:**
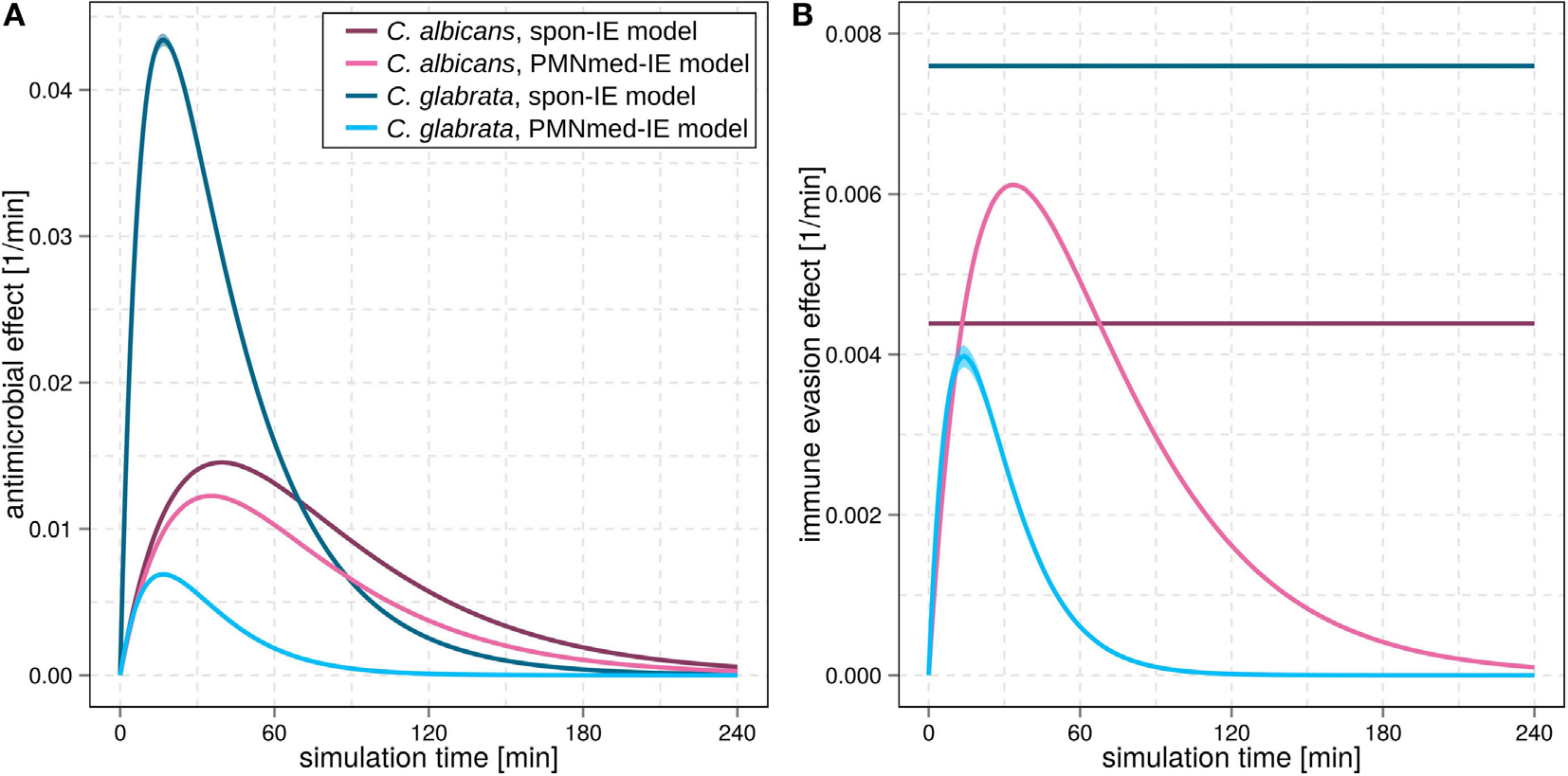
Kinetics of the extracellular killing rate **(A)** and immune-evasion rate **(B)** predicted by spon-IE model and PMNmed-IE model. In both subfigures, purple lines represent results of infection with *Candida albicans* and blue lines depict results of infection with *Candida glabrata*. Predictions by the spon-IE model and PMNmed-IE model are indicated by dark colored lines and pale colored lines, respectively.

For *C. albicans* infection, the comparison between the spon-IE model and the PMNmed-IE model revealed comparable values for most transition rates, such as ϕ*_N_*, ϕ*_M_*, κ*_N_*, and ${\bar{\kappa }}_{EK}$ (see Figure 2C; Table S2 in Supplementary Material). The largest differences were observed for intracellular killing in monocytes $\left(\kappa_{M}^{\text{PMNmed}-\text{IE}}/\kappa_{M}^{\text{spon}-\text{IE}}=1.66\right)$ and the decrease of the antimicrobial effect $\left(\gamma^{\text{PMNmed}-\text{IE}}/\gamma^{\text{spon}-\text{IE}}=1.26\right)$. However, the whole-blood infection assay does not allow to directly measure differences in these values in order to distinguish between the two immune-evasion models. Similarly, quantitative differences could also be observed for the kinetics of extracellular killing due to antimicrobial peptides (see Figure 3A) as well as for the kinetics of immune evasion (see Figure 3B). However, these readouts of the simulations either yield only small quantitative differences (time-dependent killing by antimicrobial peptides) or are, despite the qualitatively different time course, again not directly accessible in experiment (time-dependent immune-evasion rate). Thus, while it is possible to reconcile both models with the experimental data, differences in directly measurable quantities could not be identified (see Figures S3 and S4 in Supplementary Material for *C. albicans*).

While the experimental kinetics for *C. glabrata* infection were also found to be in excellent agreement with both the spon-IE and the PMNmed-IE models (see Figures 2D,E), differences between the estimated transition rate values were relatively large with up to 23% (see Figure 2F; Table S3 in Supplementary Material). The time-dependent extracellular killing due to antimicrobial factors was found to be strongly different between the two models, i.e., the peak values were six times higher for spon-IE model than PMNmed-IE model (see Figure 3A) and also the kinetics of immune-evasion were indicative for a larger effect in the spon-IE model than the PMNmed-IE model (see Figure 3B). The amount of fungal cells that became immune-evasive increased until 45 min post infection and then leveled off at the predicted value 7.47 ± 0.58% in the spon-IE model and 4.09 ± 1.0% in the PMNmed-IE model.

The comparison of whole-blood infections with the two pathogens revealed the estimated phagocytosis rate values ϕ*_N_* and ϕ*_M_* to be in both immune-evasion models lower for *C. albicans* than the phagocytosis rates of *C. glabrata*. Furthermore, for *C. albicans*, we found that ϕ*_N_* > ϕ*_M_*, whereas this relation is reversed for *C. glabrata*, reflecting the observed higher association of this pathogen to monocytes. Interestingly, the spon-IE model for infection with *C. glabrata* in comparison to infection with *C. albicans* predicted a higher peak value of the antimicrobial effect by a factor three (see Figure 3A). In contrast, the PMNmed-IE model predicted a peak value of the antimicrobial effect that is lower by a factor 0.5 for infection with *C. glabrata* compared to *C. albicans*. Apart from these observations, the two immune-evasion models could equally well explain the experimental kinetics of infection in whole-blood samples as obtained from the healthy blood donors. To work out differences between the two immune-evasion models, we addressed the question how the models differ in their predictions on the infection kinetics for virtual patients with varying severity levels of neutropenia.

### Simulations for Virtual Patients With Neutropenia Reveal Differences Between Immune-Evasion Models

**Figure 4:**
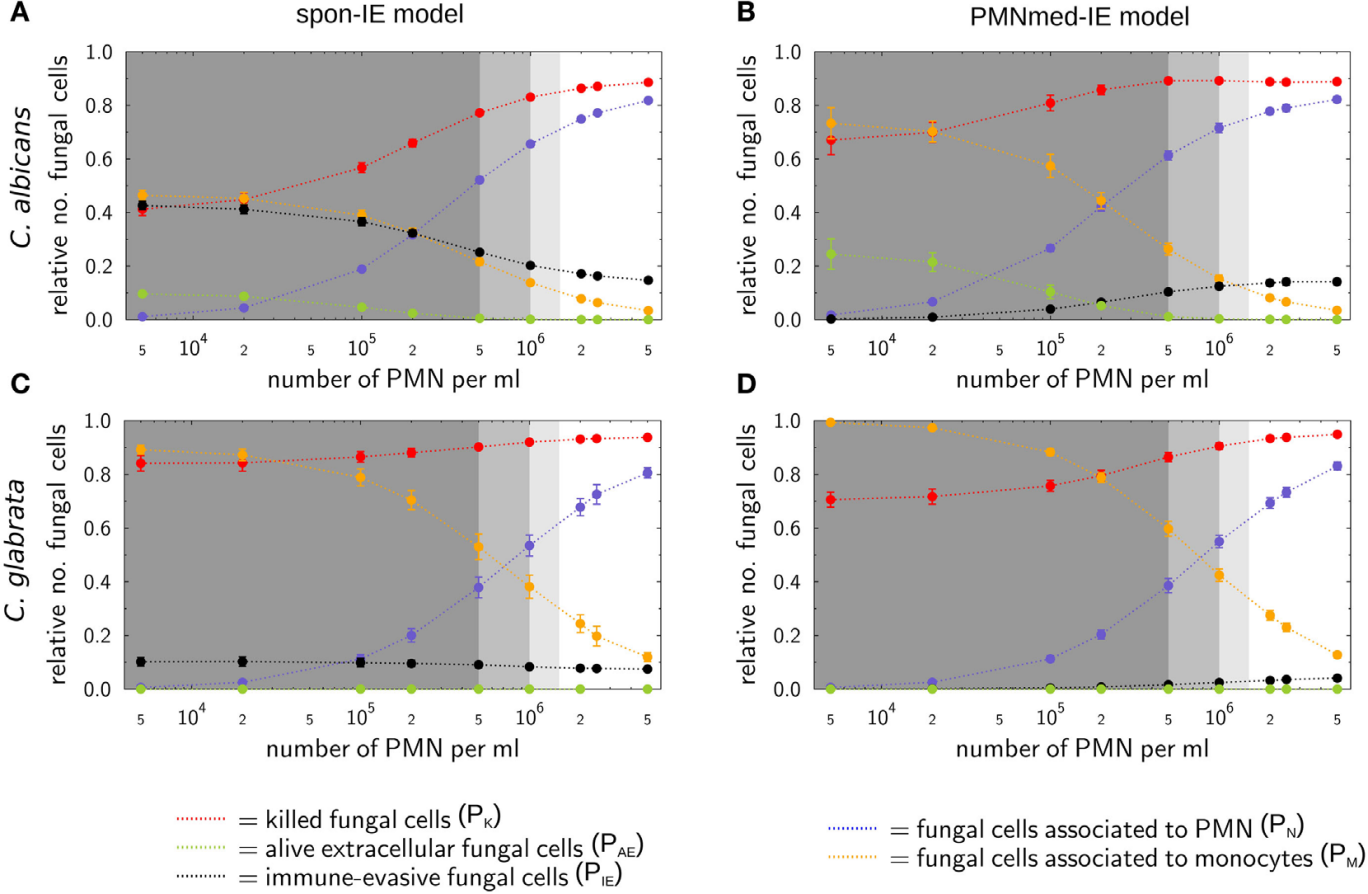
Simulation results of the spon-IE model and of the PMNmed-IE model for different severity levels of neutropenia at the time point 240 min. The white region represents the physiological concentration of a whole-blood sample with 5⋅10^5^ monocytes per milliliter and 5 ⋅10^6^ PMN per milliliter. The PMN concentration declines with increasing severity levels of neutropenia: light gray area represents mild neutropenia (<1.5⋅10^6^ PMN/ml), medium gray area represents moderate neutropenia (<1⋅10^6^ PMN/ml), and dark gray area represents severe neutropenia (<5⋅10^5^ PMN/ml). The error bars indicate SDs of 50 simulations with transition rate values that were randomly sampled within their corresponding SD. **(A,B)** Depict simulation results of a virtual *Candida albicans* infection, respectively, for the spon-IE model and of the PMNmed-IE model and **(C,D)** accordingly for *Candida glabrata* infection. The relative numbers of killed fungal cells (red), alive extracellular fungal cells (green), phagocytosed fungal cells by monocytes (yellow), and by PMN (blue), as well as fungal cells which evaded the immune defense (black) are depicted. Note that alive extracellular cells do not comprise alive immune-evasive cells and that these combined units are excluding each other.

The main difference between the spon-IE model and the PMNmed-IE model is that immune evasion in the latter is mediated by PMN and, therefore, is directly associated with the number of PMN in whole blood. Although most patients with candidemia are non-neutropenic, it is well known that neutropenia results in an impaired prognosis and facilitates disseminated infection and organ manifestation (16). Taking the previously estimated transition rate values for healthy blood donors as a reference, we gradually decreased the PMN number in the simulations within the range of medically established severity levels of neutropenia (see Table 1) and kept the number of monocytes and fungal cells fixed at 5 ⋅10^5^ cells and 1 ⋅10^6^ cells per milliliter, respectively. The predictions of simulations at 4 h post infection for the two immune-evasion models and for each of the two fungal pathogens are shown in Figure 4. As could be expected, an increase in the severity level of neutropenia was accompanied by a decreased interaction of fungal cells with PMN.

**Figure 5:**
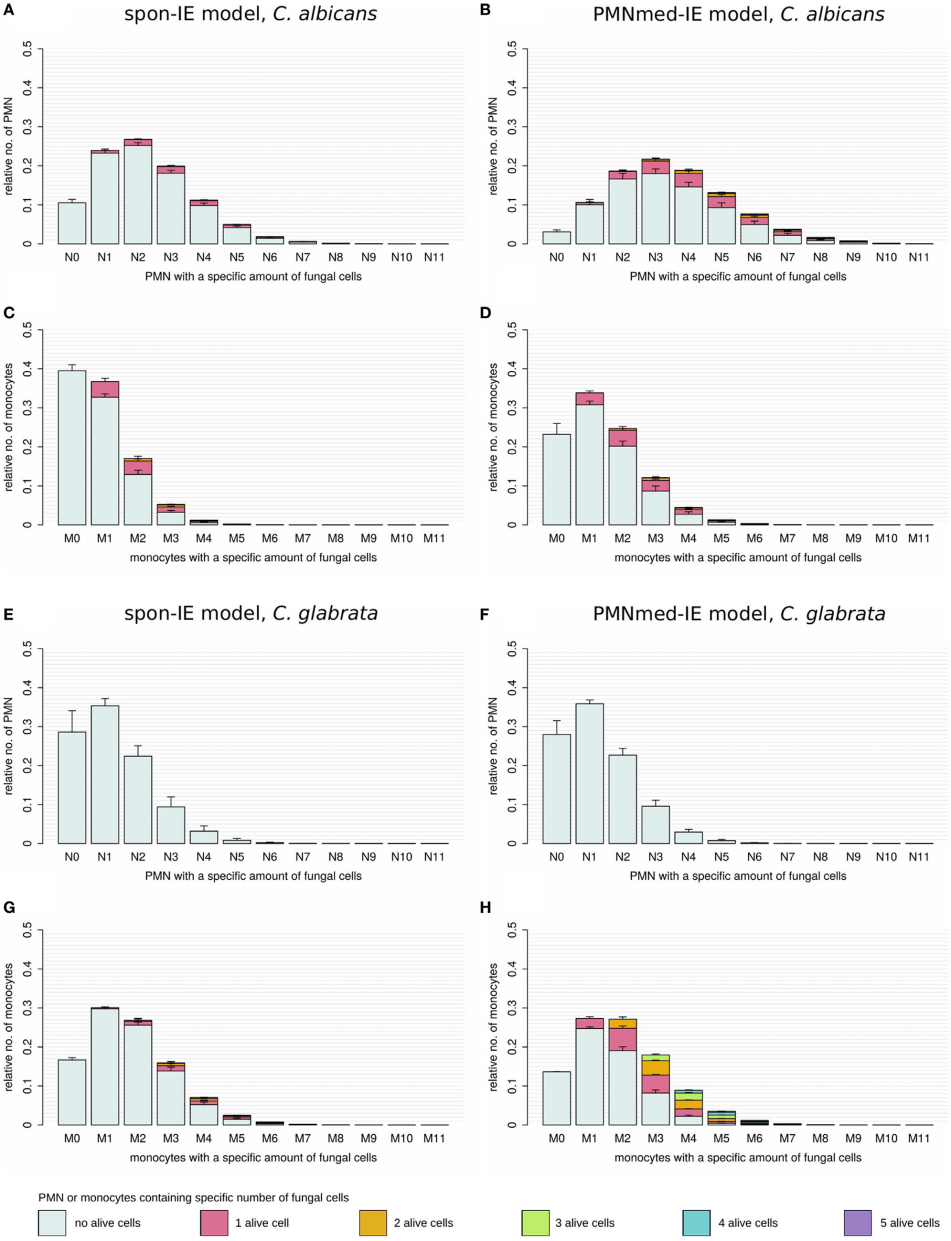
Distribution of total and alive fungal cells in PMN and monocytes for the most severe neutropenic condition 500 PMN/ml for the spon-IE model (left column) and PMNmed-IE model (right column). Relative numbers of PMN and monocytes are depicted corresponding to their association with fungal cells while each bar represents the immune cell type with the total number (0–11) of phagocytosed fungal cells. The error bars refer to SDs of 50 simulations with transition rate values that were randomly sampled within their corresponding SD. Gray-colored bars refer to “no alive” fungal cells, i.e., phagocytes contain killed cells only, bars in pink color refer to phagocytes with one alive fungal cell, orange bars refer to two alive fungal cells, green bars refer to phagocytes with three alive fungal cells, blue bars refer to phagocytes with four alive fungal cells, and purple bars refer to phagocytes with five alive fungal cells. **(A–D)** *Candida albicans* cell distribution for a virtual infection under the condition of severe neutropenia. **(E–H)** *Candida glabrata* cell distribution for a virtual infection under the condition of severe neutropenia.

Virtual infections with *C. albicans* cells under neutropenic conditions revealed clear differences between the spon-IE model (see Figure 4A) and the PMNmed-IE model (see Figure 4B) at 240 min post infection. Differences in the models could be observed at the transition from moderate to severe neutropenia, where the fraction of immune-evasive fungal cells increased to 25.2 ± 1.0% in the spon-IE model and decreased to 10.4 ± 1.1% in PMNmed-IE model. These values for immune-evasive cells changed to 42.7 ± 1.6% for the spon-IE model and 0.24 ± 0.03% for the PMNmed-IE model in the simulations with the lowest PMN number (5 ⋅10^3^ cells/ml). Even though the latter immune-evasion model predicted the number of immune-evasive *C. albicans* cells after 240 min post infection to be vanishingly small, the fraction of extracellular alive fungal cells was larger with 24.5 ± 5.6% for the PMNmed-IE model than for the spon-IE model with 9.7 ± 1.1%. In the simulations with the lowest PMN number, the spon-IE model predicted an association of 46.4 ± 1.9% fungal cells to monocytes, which is clearly lower compared to 73.3 ± 5.8% in the PMNmed-IE model. Furthermore, the number of killed *C. albicans* cells differs between the two models with being predicted as 41.2 ± 2.3% in the spon-IE model and 67.1 ± 5.5% in the PMNmed-IE model. In general, we observed that the differences in various fractions of *C. albicans* cells between the two immune-evasion models clearly increase with progressing simulation time under neutropenic conditions. This can be seen in Video S1 in the Supplementary Material showing the development of the various fungal cell fractions at specific time points between time point 0 and 240 min post infection. Furthermore, differences between the models were observed for the distribution of fungal cells in immune cells for the condition of severe neutropenia with 5 ⋅10^3^ PMN per milliliter. As shown in (Figures 5A,B), the distribution of alive and killed fungal cells across immune cells revealed differences between the immune-evasion models. Here it can be seen that the maximum of the distribution refers to PMN that contain two *C. albicans* cells for the spon-IE model (see Figure 5A) and three *C. albicans* cells for the PMNmed-IE model (see Figure 5B). Regarding the distribution of fungal cells in monocytes, the spon-IE model and the PMNmed-IE model predicted that the maximum number of monocytes which contained no fungal cells (see Figure 5C) and one fungal cell (see Figure 5D), respectively. These differences are accompanied by an overall shift of the distributions to higher numbers of phagocytes with more fungal cells in the PMNmed-IE model relative to the spon-IE model (see Figures 5A–D). In addition, the spon-IE model predicted a fraction of 7.0 ± 0.5% PMN that contain alive *C. albicans* cells (see Figure 5A), whereas this fraction of PMN was predicted to be more than two times larger in the PMNmed-IE model (19.9 ± 1.5%) (see Figure 5B).

Simulations for *C. glabrata* infection revealed as well differences between the spon-IE model and the PMNmed-IE model (see Figures 4C,D). The fraction of immune-evasive cells attained the value 10.2 ± 1.6% for the spon-IE model and 0.02 ± 0.00% for the PMNmed-IE model in the limit of lowest PMN number (5 ⋅10^3^ cells/ml). While these fractions reached different values, the fractions of extracellular alive cells were found to be vanishingly small in both models. At the PMN number of 5 ⋅10^3^ cells/ml, the spon-IE model predicted 84.1 ± 1.6% of *C. glabrata* cells to be killed and the majority of cells were phagocytosed by monocytes (89.2 ± 1.7%). Analysis of simulations of the PMNmed-IE model revealed that 70.6 ± 2.8% of *C. glabrata* cells were killed and the majority of cells were phagocytosed by monocytes (99.3 ± 0.06%). The time courses of each of these *C. glabrata* fractions at specific time points between 0 and 240 min post infection are shown in Video S2 in the Supplementary Material. Here it can be seen that at early time points post infection, the differences between the immune-evasion models is clearly visible. But with increasing simulation time these differences become smaller. While the distribution of killed and alive *C. glabrata* cells in PMN was similar for both immune-evasion models (see Figures 5E,F), differences in the distributions of fungal cells in monocytes, and their state of viability were observed (see Figures 5G,H). As can be seen in Figure 5G, the spon-IE model predicted that monocytes contained one to six fungal cells, where only a small fraction of fungal cells was alive, i.e., up to 7.1 ± 0.9% of monocytes contained alive fungal cells. This is in contrast to the PMNmed-IE model (see Figure 5H), which predicted a four times larger fraction of monocytes containing alive fungal cells (31.7 ± 1.0%). Thus, under severe neutropenic conditions, the most remarkable differences between the immune-evasion models were obtained with regard to the distribution of alive *C. glabrata* cells in monocytes.

Taken together, comparing the simulations of virtual patients under neutropenic conditions for the two immune-evasion models revealed, except for the number of immune-evaded cells, a qualitative agreement for both pathogens (see Figure 4). Comparing the infection outcome between the two pathogens for each immune-evasion models revealed qualitative agreement, except for the alive extracellular fungal cells that increase (decrease) in the case of *C. albicans* (*C. glabrata*) with higher severity levels of neutropenia. As previously observed for whole blood from healthy donors, the fraction of immune-evasive cells for neutropenic patients was predicted to be higher for *C. albicans* than for *C. glabrata* in the spon-IE model. In contrast, the PMNmed-IE model predicted for both pathogens a quantitatively comparable fraction of immune-evasive cells that vanishes with the severity level of neutropenia. The phagocytosis by monocytes was found to be much lower for *C. albicans* than for *C. glabrata*, for both immune-evasion models, as previously observed for whole blood from healthy donors. This observation was also reflected by the distribution of fungal cells in immune cells (see Figure 5). *C. glabrata* was also represented by relatively large numbers of alive cells in monocytes at 4 h post infection. These findings indicate that infection in neutropenic whole blood could shed light on the mechanism of immune evasion by pathogens.

## Discussion

In this study, we applied mathematical modeling to investigate the yet unresolved mechanism of immune evasion by pathogens in human blood. The mechanism of immune evasion was first described in a systems biology study that quantified the immune response to *C. albicans* in human whole blood using a state-based virtual infection model (12, 13). Since the mechanism of immune evasion has not been identified so far, the immune evasion was assumed to occur spontaneously with a time-independent rate in the SBM (spon-IE model). In this study, we modified the spon-IE model by implementing a time-dependent immune-evasion mechanism mediated by PMN and refer to this virtual infection model as PMNmed-IE model. This is based on experimental findings, which show that neutrophils can modulate the composition of the fungal cell surface (15). The state-based modeling approach enables realization of such a process by a transition rate that is time-dependent and reflects PMN dynamics of phagocytosis and release of neutrophilic peptides. In order to verify the PMNmed-IE model and the spon-IE model, we estimated the *a priori* unknown transition rates of these models by fitting the simulated kinetics to the experimental data from human whole-blood infection assays with either *C. albicans* or *C. glabrata*. To further work out differences between the immune-evasion models, we simulated infection scenarios with reduced numbers of PMN that correspond to the range of medically established severity levels of neutropenia.

The comparison of the simulated kinetics for infections of blood with physiological and reduced numbers of PMN, the estimated transition rate values, as well as the pathogen distribution across immune cells revealed pathogen-specific differences between the two immune-evasion models. Based on these results, we suggest future experiments that could be performed to distinguish between the two immune-evasion mechanisms. While the kinetics of the experimental whole-blood infection assays for both pathogens could be reconciled with the virtual infection kinetics for both immune-evasion models, simulations for reduced PMN numbers revealed differences between the two immune-evasion models. These differences were largest for *C. albicans* infection and relatively small for infections with *C. glabrata*. In particular, the fractions of fungal cells that were killed, associated with monocytes or that became immune-evasive in simulations with reduced numbers of PMN, showed deviations between the two immune-evasion models most clearly for *C. albicans* (see Figures 4A,B). With decreasing PMN number, the PMNmed-IE model for this pathogen predicted that the fraction of immune-evasive pathogens remarkably decreased. Instead of becoming immune-evasive, *C. albicans* cells were mainly phagocytosed by monocytes and killed in this model. Furthermore, a significant fraction of fungal cells (24.5 ± 5.6%) was still alive and in extracellular space at 240 min post infection. In contrast to the PMNmed-IE model, the spon-IE model predicted the fractions of *C. albicans* cells that are (i) phagocytosed by monocytes, (ii) killed, or (iii) remained viable in extracellular space to be notably smaller, whereas the fraction of immune-evasive *C. albicans* cells is larger, because the constant rate of immune evasion does not depend on the decreasing number of PMN. Interestingly, both immune-evasion models predict even at 240 min post infection a remarkable fraction of *C. albicans* cells that are capable of dissemination. However, in the PMNmed-IE model these cells are mainly alive and extracellular due to absent phagocytosis whereas in the spon-IE model they are mostly immune-evasive fungal cells. Thus, both models would explain the observation that dissemination of *C. albicans* is more frequent in a neutropenic setting, albeit with different mechanisms (26–28). In order to verify the predicted differences for the two immune-evasion models, we suggest studying whole-blood infection assays either with depleted PMN numbers or with blood samples from neutropenic patients.

Regarding the pathogen distribution across immune cells, virtual infection scenarios for *C. albicans* with the low PMN number of 5 ⋅10^3^ cells/ml revealed differences between the two immune-evasion models in the pathogen distributions within PMN and monocytes as well as in the fraction of alive *C. albicans* cells in PMN (see Figures 5A–D). The experimental validation of the pathogen distribution in PMN and monocytes could be performed by Giemsa-stained blood smears obtained from *C. albicans*-infected blood samples of neutropenic patients. The overall distribution of *C. albicans* cells in PMN and monocytes could lead to further conclusions by comparing the experimental observations to simulated results, although information about viability cannot be obtained by Giemsa-stained blood smears. For the experimental validation of pathogen distribution across immune cells during infection of neutropenic blood samples it is necessary to differentiate between alive and killed fungal cells to unravel the immune-evasion mechanism of *C. glabrata*. The virtual infection of neutropenic blood by this pathogen showed clear differences between the immune-evasion models with regards to the distribution of alive pathogen cells in monocytes (see Figures 5G,H). The PMNmed-IE model predicted a relatively large fraction of alive fungal cells in monocytes at 240 min post infection. With increasing infection time in neutropenic patients, the high amount of alive fungal cells in monocytes may result in higher amounts of fungal cells in macrophages, which are professional phagocytes of the monocytic lineage. Since it is reported that *C. glabrata* cells are able to proliferate within macrophages and subsequently can leave these phagocytes (21, 29), this process could contribute to the increased risk for disseminated candidiasis in neutropenic patients (30).

Another suggestion for the experimental investigation of the immune-evasion mechanisms is to measure the activity of antimicrobial effector proteins inducing extracellular killing, because these kinetics are predicted to be different for the two immune-evasion models. This difference was observed to be relatively high for virtual *C. glabrata* infection at physiological numbers of PMN: in the spon-IE model the maximum value for the extracellular killing rate was much larger for *C. glabrata* infection compared to *C. albicans*, whereas in the PMNmed-IE model this peak value was predicted to be much smaller for *C. glabrata* infection (see Figure 3A). We, therefore, suggest measuring and comparing the activity of antimicrobial effector proteins inducing extracellular killing, such as lactoferrin, elastase 2 and myeloperoxidase, for both pathogens. In a previous study by Duggan et al. (25), where the differential recognition of *C. albicans* and *C. glabrata* by PMN was investigated, the concentration of these proteins were measured in supernatants of confrontation assays of PMN with the fungi 4 h after infection. For each of these antimicrobial proteins, the concentration in confrontation assays with *C. albicans* was observed to be higher than in confrontation assays with *C. glabrata*. We now suggest measuring not only the concentration of these antimicrobial peptides but also their fungicidal effect on the different pathogens in a comparative fashion. Moreover, our analysis predicts the time-window, where the largest difference for the kinetics of extracellular killing between both pathogens occurs, i.e., at 10 to 50 min post infection.

In future studies, the predictive power of virtual infection modeling can be further exploited by simulating infection scenarios with modified models that enable generating predictions for other hypotheses. For example, while the present study focused on the role of PMN-mediated immune evasion, a similar mechanism could be studied for monocytes, as well as a combination of contributions from both types of immune cells. Future computational studies could also benefit from spatial agent-based modeling. By applying a bottom-up approach, as previously performed by Lehnert et al. (13), the transition rate values of the SBM could be used as input for an agent-based model, where also spatial system properties are captured, such as the cells’ morphology and/or migration pattern. This agent-based virtual infection model could, for example, be applied to investigate the impact of the various immune-evasion models on a hyper- and hypo-inflammatory immune response in human blood. In addition, the impact of the spatial distribution of PMN-secreted proteins causing immune evasion could be investigated by advancing the cellular agent-based virtual infection model to a hybrid agent-based model that simulates diffusion at the molecular level by partial differential equations. For example, in previous studies related to fungal infections, a hybrid agent-based model enabled to investigate the immune response against *Aspergillus fumigatus* in the alveoli of the human lung (31, 32). It could be shown that the migration pattern of immune cells is of high importance for the timely infection clearance and this lead to the prediction that chemotactic signaling molecules are essential for recruitment of phagocytes to the spatial position of fungal cells in the lung. Moreover, image-based systems biology approach combining mathematical modeling with microscopy experiments could be pursued (9, 33, 34). While imaging in whole blood is not performed today, host–pathogen interactions can be investigated by microscopy experiments under controlled conditions in a Petri dish. Recently, we have developed algorithms for the fully automated analysis of host–pathogen confrontation from microscopic endpoint experiments (33, 35–38), as well as from live cell imaging (39, 40). Similar to our recent comparative studies on *C. albicans* and *C. glabrata* phagocytosis (16, 41), host–pathogen confrontation assays could be performed and analyzed by automated image analysis to visualize surface alterations of immune-evading fungal cells.

## Ethics Statement

This study was conducted according to the principles expressed in the Declaration of Helsinki. All protocols were approved by the Ethics Committee of the University Hospital Jena (permit number: 273-12/09). Written informed consent was obtained from all blood donors.

## Author Contributions

Conceived and designed this study: TL and MF. Provision of computational resources: MF. Provision of materials: OK. Data processing, implementation, and application of the computational algorithm: MP, TL, and MF. Performed experiments: KH and IL. Evaluation and analysis of the results: MP, TL, ST, KH, IL, OK, and MF. Draft and revision of the manuscript: MP, TL, ST, KH, IL, OK, and MF.

## Conflict of Interest Statement

The authors declare that the research was conducted in the absence of any commercial or financial relationships that could be construed as a potential conflict of interest.

## Supplementary Material

Video S1 Time-course of simulation results of both immune-evasion models for *C. albicans* infection for different severity levels of neutropenia. The error bars indicate standard deviations of 50 simulations with transition rate values that were randomly sampled within their corresponding standard deviation. The white region represents the physiological concentration of a whole-blood sample with 5⋅10^5^ monocytes per milliliter and 5⋅10^6^ PMN per milliliter. The PMN concentration declines with increasing severity levels of neutropenia: light gray area represents mild neutropenia (<1.5⋅10^6^ PMN/ml), medium gray area represents moderate neutropenia (<1⋅10^6^ PMN/ml) and dark gray area represents severe neutropenia (<5⋅10^5^ PMN/ml). **(A,B)** depict simulation results of a virtual *C. albicans* infection, respectively, for the spon-IE model and of the PMNmed-IE model. The relative numbers of killed fungal cells (red), alive extracellular fungal cells (green), phagocytosed fungal cells by monocytes (yellow) and by PMN (blue), as well as fungal cells which evaded the immune defense (black) are depicted. Note that alive extracellular cells do not comprise alive immune-evasive cells and that these combined units exclude each other.

Video S2 Time-course of simulation results of both immune-evasion models for *C. glabrata* infection for different severity levels of neutropenia. The error bars indicate standard deviations of 50 simulations with transition rate values that were randomly sampled within their corresponding SD. The white region represents the physiological concentration of a whole-blood sample with 5⋅10^5^ monocytes per milliliter and 5⋅10^6^ PMN per milliliter. The PMN concentration declines with increasing severity levels of neutropenia: light gray area represents mild neutropenia (<1.5⋅10^6^ PMN/ml), medium gray area represents moderate neutropenia (<1⋅10^6^ PMN/ml) and dark gray area represents severe neutropenia (<5⋅10^5^ PMN/ml). **(A,B)** depict simulation results of a virtual *C. glabrata* infection, respectively, for the spon-IE model and of the PMNmed-IE model. The relative numbers of killed fungal cells (red), alive extracellular fungal cells (green), phagocytosed fungal cells by monocytes (yellow) and by PMN (blue), as well as fungal cells which evaded the immune defense (black) are depicted. Note that alive extracellular cells do not comprise alive immune-evasive cells and that these combined units exclude each other.
