# Supplementary material for: Predictive Virtual Infection Modeling of Fungal Immune Evasion in Human Whole Blood
Source: Front Immunol. 2018 Mar 21;9:560. doi: 10.3389/fimmu.2018.00560 (PMC5871695; doi:10.3389/fimmu.2018.00560)
Supplement: Supplementary file 3 [file data_sheet_1.PDF]

Supplementary Material

Predictive Virtual Infection Modeling of Pathogen Immune Evasion in Human Whole Blood

Maria T. E. Prauße, Teresa Lehnert, Sandra Timme, Kerstin Hünniger, Ines Leonhardt, Oliver Kurzai, Marc Thilo Figge*

*** Correspondence:** Marc Thilo Figge: thilo.figge@leibniz-hki.de

1 Supplementary Data

1.1 Supplementary Information

S1. Simulation algorithm of the state-based whole-blood infection model.

The temporal evolution of the state-based model (SBM) within the simulation time $t = 0, \dots, t_{end}$ is calculated for each simulation time step Δt (with $\Delta t = 1$ min) by updating all individuals of the system with respect to their current state. In particular, an individual of state *s* will be updated by testing for transition into another state *s'*. Each state transition is associated with a rate, $r^{s \to s'}$, and will be executed with probability $P_{s \to s'} = r^{s \to s'} \Delta t$. The system is updated accordingly. As schematically depicted in Figure S1 in Supplementary Material, the simulation of the SBM for whole-blood infection comprises updating individuals of the states P_{AE} and P_{KE}, which are respectively alive extracellular pathogens and killed extracellular pathogens, as well as individuals of the states $M_{i,j}$ and $N_{i,j}$, which respectively represent monocytes and neutrophils containing i alive and j killed pathogens. Individuals of the states P_{AE} and P_{KE} are tested for extracellular killing and immune evasion, whereas individuals of the states $M_{i,j}$ and $N_{i,j}$ are tested for phagocytosis and intracellular killing. A description of the transition rates of the SBM and the respective state transitions are given in Table S1 in Supplementary Material.

This SBM simulation algorithm was implemented in the programming language C++ and is available for download from https://asbdata.hki-jena.de/publidata/PrausseEtAl2018_FrontImmunol/ .

S2. Parameter estimation by the global optimization method Simulated Annealing based on the Metropolis Monte Carlo scheme.

By randomly exploring the parameter space of transition rates used in the spon-IE model and PMNmed-IE model, the algorithm is able to find the global minimum of the least-square error (LSE) between the simulated data of the models and the experimental data acquired from the whole-blood infection assays. The flowchart of this algorithm is depicted in Figure S2 in Supplementary Material.
The time-evolution of the state-based model kinetics, referred to as *combined units*, is simulated using parameter set $\vec{p}$ and compared with the experimental data by calculating the weighted sum of LSE ($E(\vec{p})$). This is followed by choosing a parameter set $\vec{p}'$ within a pre-defined range of ±10% from $\vec{p}$. Next, the model is simulated with the parameter set $\vec{p}'$ and the corresponding score $E(\vec{p}')$ is calculated. If $E(\vec{p}')$ attains a smaller value than $E(\vec{p})$, the new parameter set $\vec{p}'$ is accepted because of

the better fitting result. However, $\vec{p}'$ may also be accepted for $E(\vec{p}')$ larger than $E(\vec{p})$ with a probability that depends on the Boltzmann distribution (Metropolis Monte Carlo Step, see Step 3 in Figure S2 in Supplementary Material). This probability is decreased with progressing number of fitting steps f, simulating the system's annealing by a decreasing of temperature parameter $\tau(f)$ in the Boltzmann distribution. Thus the risk to accept an unfavorable parameter set $\vec{p}'$ decreases with the ongoing fitting procedure by the system's temperature decrease. The choice of a new parameter set $\vec{p}'$, its evaluation and acceptance or rejection is repeated for a specific number of fitting steps making up one fitting run. Fitting runs are repeated as wells starting from different initial parameter sets and the means and standard deviations of the determined optimal parameter sets resulting from these repetitions are calculated. Due to large numbers of fungal cells (P), monocytes (M) and polymorphonuclear neutrophils (N) in infected whole-blood samples, the state-based model simulation of such a system size is accompanied with large computation times. Therefore, the fitting procedure is designed in a step-wise fashion: The system size is gradually increased by factors of ten starting at $P = 100$, $M_{0,0} = 50$ and $N_{0,0} = 500$ up to $P = 10^6$, $M_{0,0} = 5 \cdot 10^5$ and $N_{0,0} = 5 \cdot 10^6$. *i.e.* keeping the ratio of cell numbers constant. Here, the optimal parameter values of a model system as determined by the above described estimation procedure serves as start values in fitting the state-based model for the next in size system.

This framework for parameter estimation of the state-based whole-blood infection model was implemented in the programming language C++ and is available for download from https://asbdata.hki-jena.de/publidata/PrausseEtAl2018_FrontImmunol/ .

1.2 Supplementary Videos

Video S1. Time-course of simulation results of both immune-evasion models for *C. albicans* infection for different severity levels of neutropenia. The error bars indicate standard deviations of 50 simulations with transition rate values that were randomly sampled within their corresponding standard deviation. The white region represents the physiological concentration of a whole-blood sample with $5 \cdot 10^5$ monocytes per milliliter and $5 \cdot 10^6$ PMN per milliliter. The PMN concentration declines with increasing severity levels of neutropenia: light gray area represents mild neutropenia ($< 1.5 \cdot 10^6\ \frac{PMN}{ml}$), medium gray area represents moderate neutropenia ($< 1 \cdot 10^6\ \frac{PMN}{ml}$) and dark gray area represents severe neutropenia ($< 5 \cdot 10^5\ \frac{PMN}{ml}$). (**A**) and (**B**) depict simulation results of a virtual *C. albicans* infection, respectively, for the spon-IE model and of the PMNmed-IE model. The relative numbers of killed fungal cells (red), alive extracellular fungal cells (green), phagocytosed fungal cells by monocytes (yellow) and by PMN (blue), as well as fungal cells which evaded the immune defense (black) are depicted. Note that alive extracellular cells do not comprise alive immune-evasive cells and that these combined units exclude each other.

Video S2. Time-course of simulation results of both immune-evasion models for *C. glabrata* infection for different severity levels of neutropenia. The error bars indicate standard deviations of 50 simulations with transition rate values that were randomly sampled within their corresponding standard deviation. The white region represents the physiological concentration of a whole-blood sample with $5 \cdot 10^5$ monocytes per milliliter and $5 \cdot 10^6$ PMN per milliliter. The PMN concentration declines with increasing severity levels of neutropenia: light gray area represents mild neutropenia ($< 1.5 \cdot 10^6\ \frac{PMN}{ml}$), medium gray area represents moderate neutropenia ($< 1 \cdot 10^6\ \frac{PMN}{ml}$) and dark gray area represents severe neutropenia ($< 5 \cdot 10^5\ \frac{PMN}{ml}$). (**A**) and (**B**) depict simulation results of a virtual *C. glabrata* infection, respectively, for the spon-IE model and of the PMNmed-IE model. The relative numbers of killed fungal cells (red), alive extracellular fungal cells (green), phagocytosed fungal cells by monocytes (yellow) and by PMN (blue), as well as fungal cells which evaded the immune defense (black) are depicted. Note that alive extracellular cells do not comprise alive immune-evasive cells and that these combined units exclude each other.

2 Supplementary Figures and Tables

2.1 Supplementary Tables

rate	description	state transition
$\boldsymbol{\phi_N}$	phagocytosis by neutrophils	$N_{i,j} + P_{AE} \rightarrow N_{i+1,j}$<br>$N_{i,j} + P_{KE} \rightarrow N_{i,j+1}$
$\boldsymbol{\phi_M}$	phagocytosis by monocytes	$M_{i,j} + P_{AE} \rightarrow M_{i+1,j}$<br>$M_{i,j} + P_{KE} \rightarrow M_{i,j+1}$
$\boldsymbol{\kappa_N}$	intracellular killing by neutrophils	$N_{i,j} \rightarrow N_{i-1,j+1}$
$\boldsymbol{\kappa_M}$	intracellular killing by monocytes	$M_{i,j} \rightarrow M_{i-1,j+1}$
$\boldsymbol{\kappa_{EK}(t)}$	extracellular killing by antimicrobial peptides released upon first-time PMN phagocytosis with decreasing activity<br><br>rate depends on the activity of antimicrobial peptides, characterized by the rate $\bar{\kappa}_{EK}$, and the decay of their antimicrobial activity, characterized by γ, as defined in Hünniger et al. (2014) (1) and Lehnert et al. (2015) (2)	$P_{AE} \rightarrow P_{KE}$
$\boldsymbol{\rho}$	constant rate for the acquisition of immune evasion for spon-IE model	$P_{AE} \rightarrow P_{AIE}$<br>$P_{KE} \rightarrow P_{KIE}$
$\boldsymbol{\rho(t)}$	time dependent rate for the acquisition of immune evasion for PMNmed-IE model<br><br>rate depends on the activity of effector molecules, characterized by $\bar{\rho}$ and the decay of their activity as characterized by γ_R (see Eq. (7) in the manuscript)	$P_{AE} \rightarrow P_{AIE}$<br>$P_{KE} \rightarrow P_{KIE}$

Table S1. Transition rates of the PMNmed-IE model and the spon-IE model for modeling the whole-blood infection assays. For details of the latter model, see Hünniger et al. (2014) (1) and Lehnert et al. (2015) (2).

C. albicans	mean $\times 10^{-2}$ $[min^{-1}]$ $\pm$ SD $\times 10^{-2}$ $[min^{-1}]$ (CV [%])	
**rate**	**spon-IE model**	**PMNmed-IE model**
$\boldsymbol{\phi_N}$	$2.97 \pm 0.061(2.1)$	$2.86 \pm 0.054(1.9)$
$\boldsymbol{\phi_M}$	1.23 ± 0.075 (6.1)	1.2 ± 0.13 (10.6)
$\boldsymbol{\kappa_M}$	2.1 ± 0.31 (14.6)	3.5 ± 0.42 (12.2)
$\boldsymbol{\kappa_N}$	5.3 ± 0.49 (9.2)	5.7 ± 0.97 (16.9)
$\boldsymbol{\bar{\kappa}_{EK}}$	21.8 ± 1.8 (8.0)	20.44 ± 3.33 (16.3)
$\boldsymbol{\gamma}$	2.13 ± 0.19 (8.8)	2.69 ± 0.34 (12.5)
$\boldsymbol{\rho}$	0.44 ± 0.021 (4.8)	
$\boldsymbol{\bar{\rho}}$		10.75 ± 0.34 (7.1)
$\boldsymbol{\gamma_R}$		3.0 ± 0.35 (11.6)

Table S2. The transition rates of a virtual whole-blood infection with *C. albicans* for the spon-IE and PMNmed-IE model are given by the phagocytosis rate ϕ_N of PMN and the phagocytosis rate ϕ_M of monocytes, the intracellular killing rates κ_M and κ_N of both monocytes and PMN, the transition rates γ and $\bar{\kappa}_{EK}$ which define the extracellular killing and the spontaneous immune evasion rate ρ and the PMN-mediated immune evasion rates $\bar{\rho}$ and γ_R, respectively. The mean values and standard deviations (SD) were obtained by several repetitions of the parameter estimation procedure which is described in the work of Lehnert et al. (2014) (1,2). CV refers to the coefficient of variation which defines the ratio of SD to the mean.

C. glabrata	mean × $\mathbf{10^{-2}}$ $[\boldsymbol{min^{-1}}]$ ± SD × $\mathbf{10^{-2}}$ $[\boldsymbol{min^{-1}}]$ (CV [%])	
rate	**spon-IE model**	**PMNmed-IE model**
$\boldsymbol{\phi_N}$	10.0 ± 0.55 (5.5)	6.5 ± 0.49 (7.6)
$\boldsymbol{\phi_M}$	14.3 ± 1.4 (9.6)	9.9 ± 0.64 (6.5)
$\boldsymbol{\kappa_M}$	2.2 ± 0.23 (10.6)	1.10 ± 0.073 (6.6)
$\boldsymbol{\kappa_N}$	4.9 ± 0.43 (8.7)	13.8 ± 2.5 (18.3)
$\boldsymbol{\bar{\kappa}_{EK}}$	47.5 ± 9.9 (20.8)	10.7 ± 1.6 (15.1)
$\boldsymbol{\gamma}$	3.1 ± 0.34 (10.9)	5.4 ± 1.3 (23.1)
$\boldsymbol{\rho}$	0.76 ± 0.047 (6.2)	
$\boldsymbol{\bar{\rho}}$		7.3 ± 1.7 (22.8)
$\boldsymbol{\gamma_R}$		7.9 ± 1.8 (22.7)

Table S3. The transition rates of a virtual whole-blood infection with *C. glabrata* for the spon-IE (left column in each split) and PMNmed-IE model (right column in each split) are given by the phagocytosis rate ϕ_N of PMN and the phagocytosis rate ϕ_M of monocytes, the intracellular killing rates κ_M and κ_N of both monocytes and PMN, the transition rates γ and $\bar{\kappa}_{EK}$ which define the extracellular killing and the spontaneous immune evasion rate ρ and the PMN-mediated immune evasion rates $\bar{\rho}$ and γ_R, respectively. The mean values and standard deviations (SD) were obtained by several repetitions of the parameter estimation procedure which is described in the work of Lehnert et al. (2014) (1,2). CV refers to the coefficient of variation which defines the ratio of SD to the mean.

2.2 Supplementary Figures

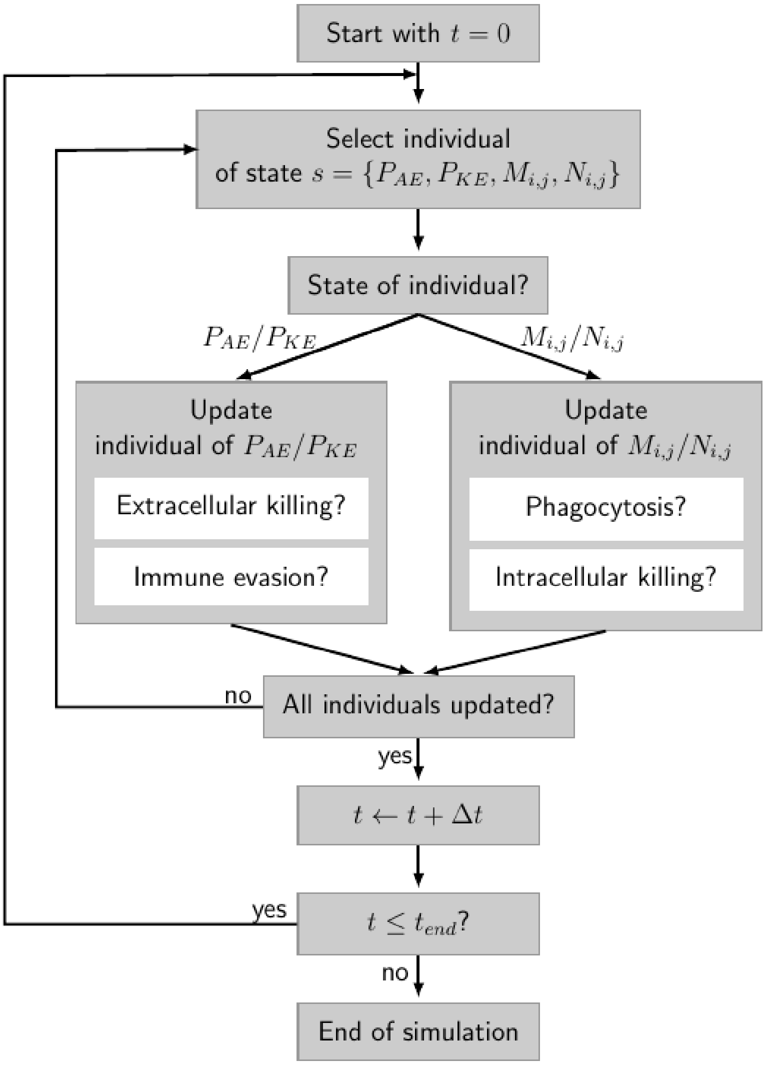

Figure S1. Flowchart of the simulation algorithm for the state-based whole-blood infection model. This algorithm is explained in detail in section S1 in Supplementary Material. The flowchart is adapted from Lehnert et al. (2015) (2).

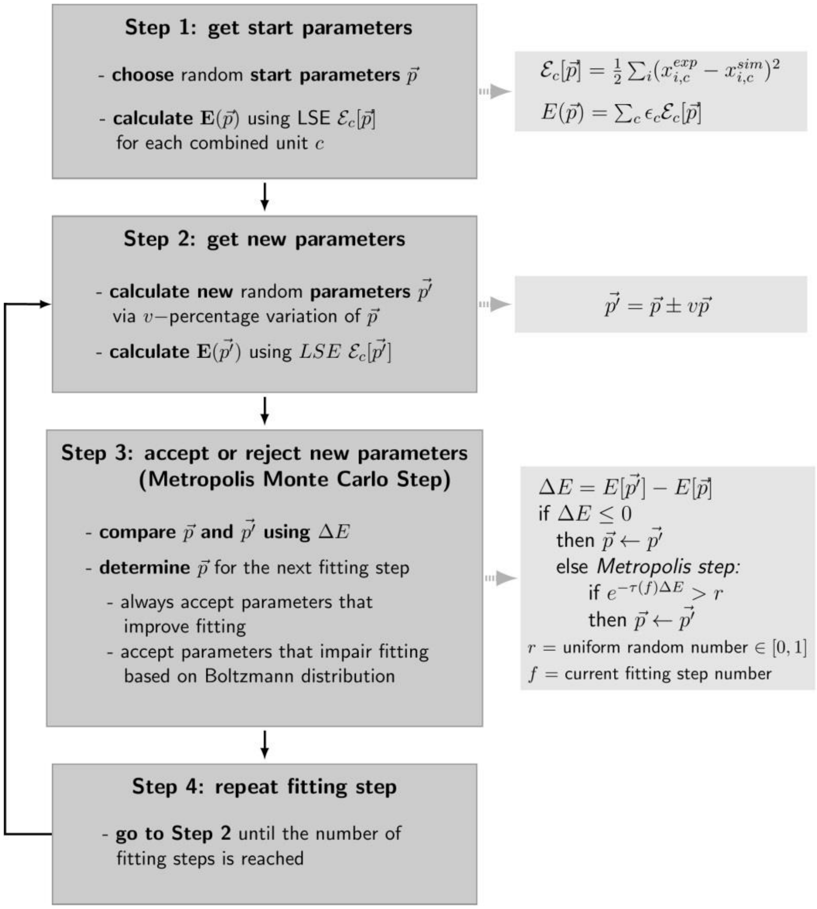

Figure 2. Flowchart of the parameter estimation algorithm. The algorithm is explained in detail in Section S2 in Supplementary Material. The flowchart is adapted from Lehnert et al. (2015) (2).

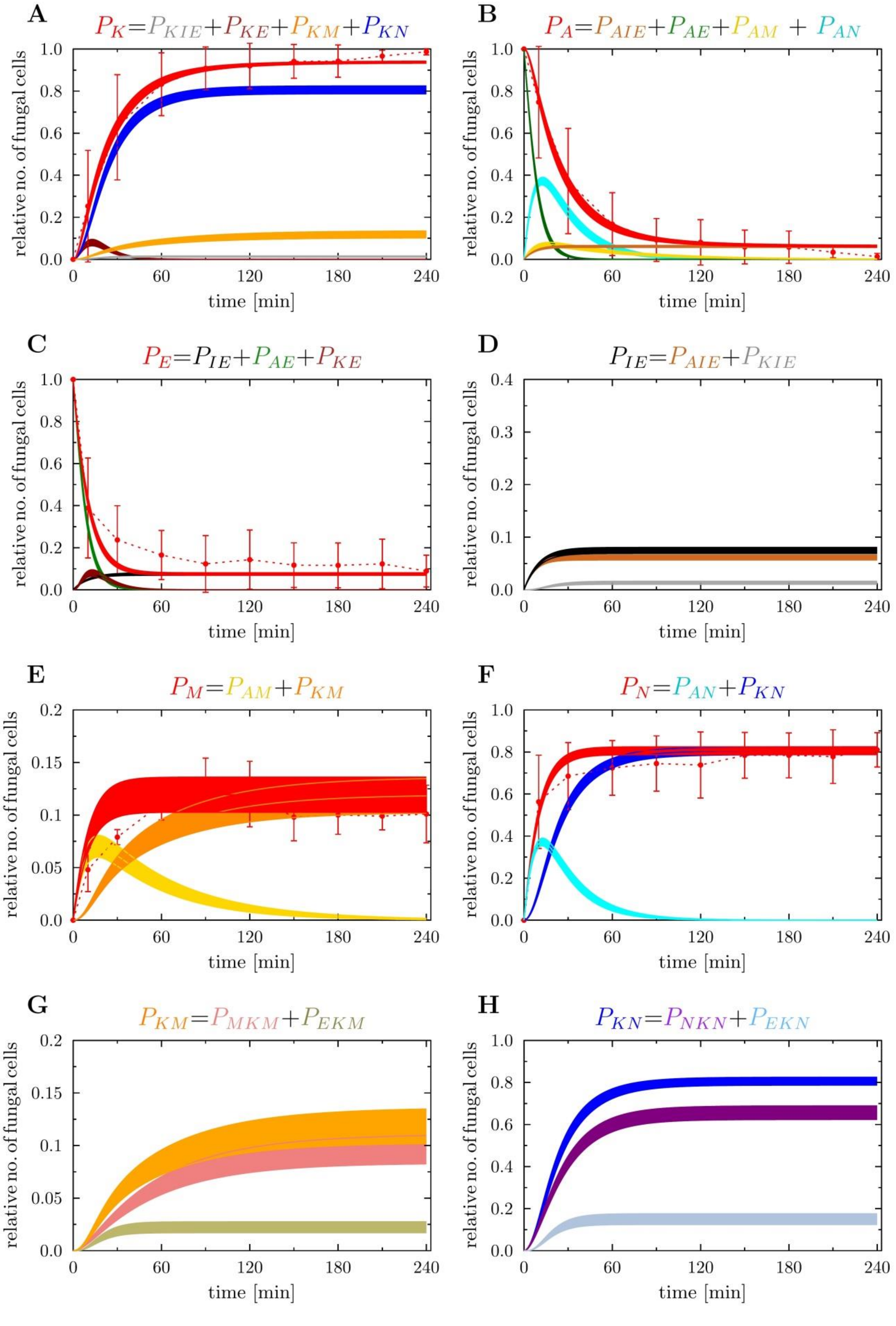

Figure S3. Kinetics of Combined Units of the spon-IE model by the use of the computed transition rates. Experimental data from whole-blood infection assays (red dotted lines) with corresponding

standard deviations are compared to the simulated data by the spontaneous evasion model. The thickness of solid lines indicates the mean $\pm$ standard deviation of 50 simulations with transition rate values that were randomly sampled within their corresponding standard deviation. The breakdown of each combined unit is depicted the following way. (**A**) Time-dependent relative number of killed *C. albicans* cells (P_K) that were experimentally measured by survival plates. The experimental results were compared with the total number of simulated killed *C. albicans* cells. P_K is defined as the sum of killed immune-evasive *C. albicans* (P_{KIE}), extracellularly killed *C. albicans* (P_{KE}), and killed *C. albicans* in monocytes (P_{KM}) and PMN (P_{KM}) (**B**) Alive *C. albicans* (P_A) were also measured by survival plates. The simulated result was then compared to the experimental data. (**C**) Time course of *C. albicans* cells that are in extracellular space in the blood. Here, experimental data was gathered via FACS analysis and compared to the simulated data. (**D**) Simulated immune-evasive *C. albicans* cells (P_{IE}) are the sum of alive and killed *C. albicans* cells, which are able to evade the immune defense, over the time course of the simulation. (**E**) Time course of the total number of alive and killed *C. albicans* cells that were phagocytosed by monocytes (P_M). (**F**) Relative number of *C. albicans* cells in PMN (P_N) during the whole-blood infection assay and comparison to the simulated result. (**G**) Simulation result of killed *C. albicans* cells within monocytes (P_{KM}), that is defined as the sum of internalized *C. albicans* that were intracellularly killed (P_{MKM}) and those who were extracellularly killed (P_{EKM}). (**H**) simulated time course of killed cells in PMN (P_{KN}), that is composed of intracellularly killed *C. albicans* cells (P_{NKN}) and extracellularly killed *C. albicans* cells (P_{EN}) in PMN.

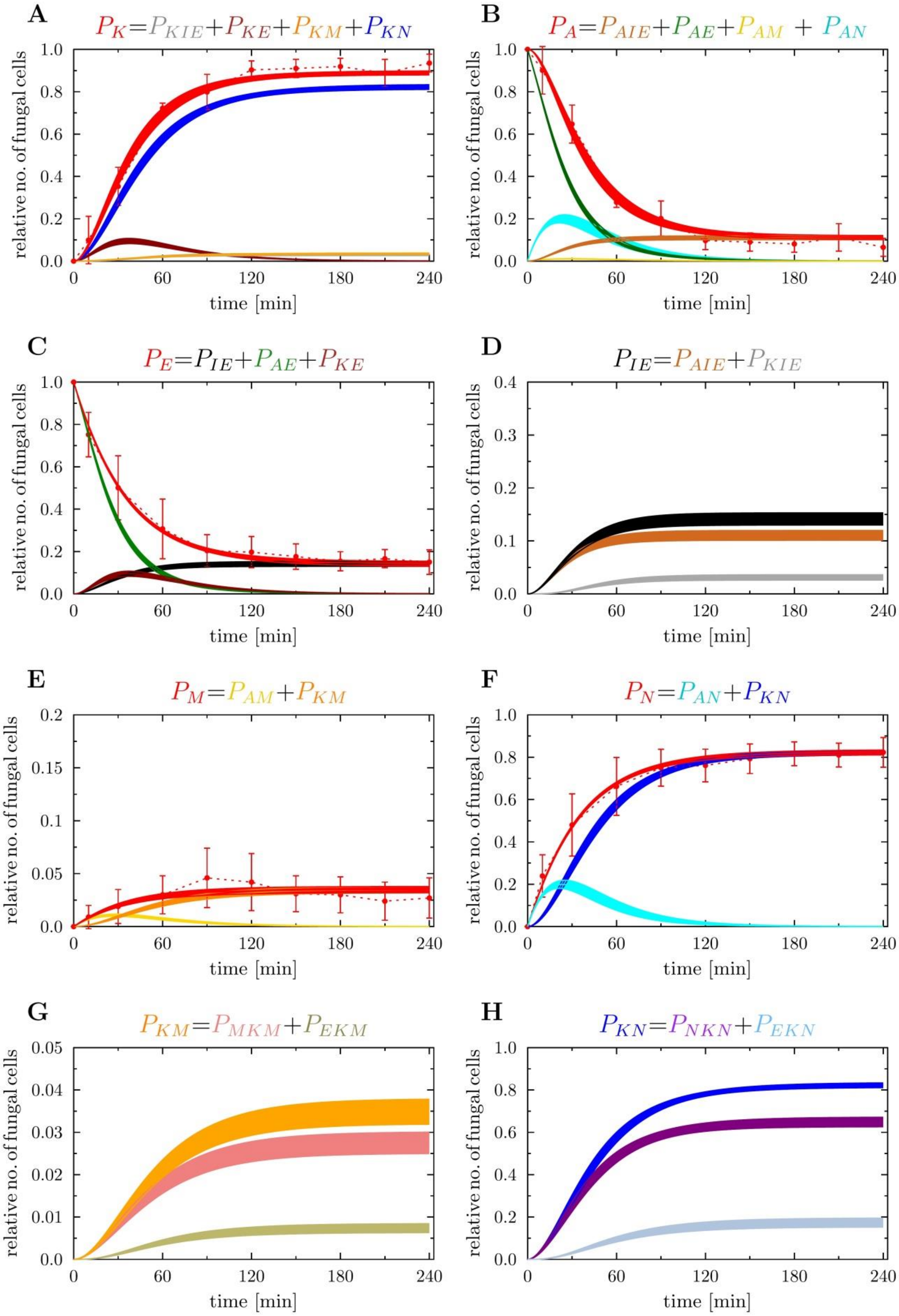

Figure S4. As in Figure S3 in Supplementary Material but for PMNmed-IE model in the case of *C. albicans* infection.

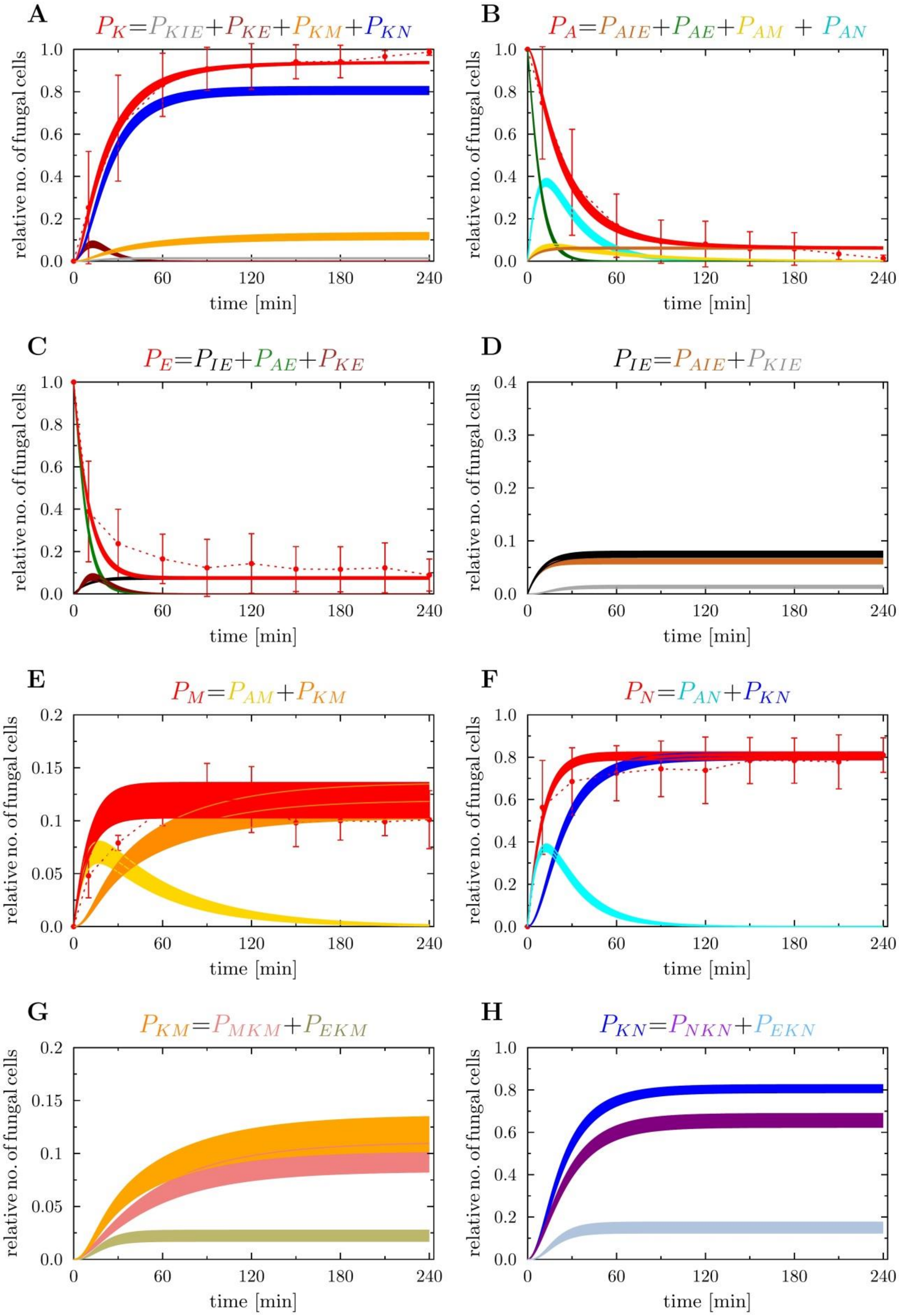

Figure S5. As in Figure S3 in Supplementary Material but for spon-IE model in the case of *C. glabrata* infection.

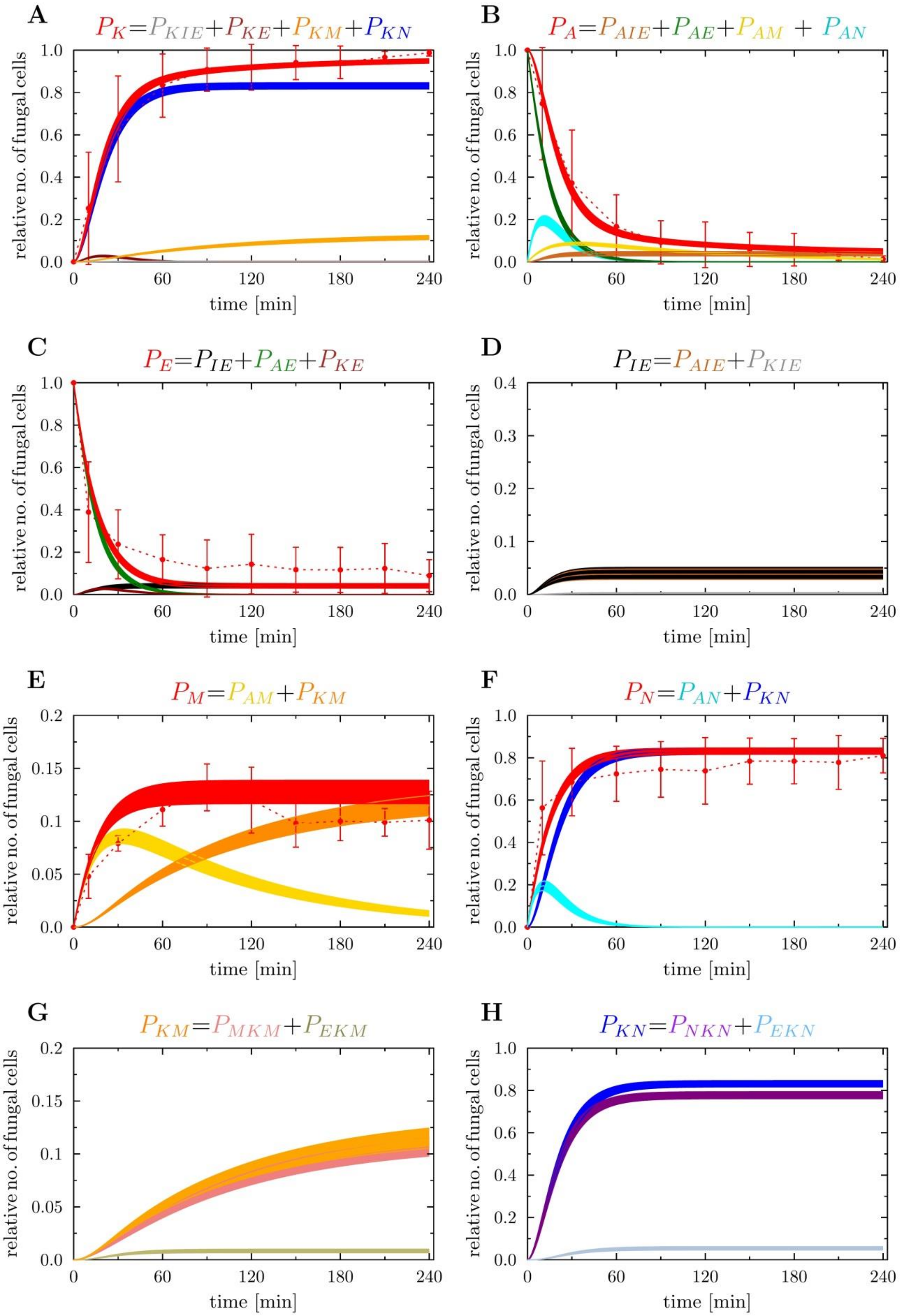
A
$P_K = P_{KIE} + P_{KE} + P_{KM} + P_{KN}$
B
$P_A = P_{AIE} + P_{AE} + P_{AM} + P_{AN}$
C
$P_E = P_{IE} + P_{AE} + P_{KE}$
D
$P_{IE} = P_{AIE} + P_{KIE}$
E
$P_M = P_{AM} + P_{KM}$
F
$P_N = P_{AN} + P_{KN}$
G
$P_{KM} = P_{MKM} + P_{EKM}$
H
$P_{KN} = P_{NKN} + P_{EKN}$
relative no. of fungal cells
time [min]
0
60
120
180
240

Figure S6. As in Figure S3 in Supplementary Material but for PMNmed-IE model in the case of *C. glabrata* infection.

3 References

1. Hünniger K, Lehnert T, Bieber K, Martin R, Figge MT, Kurzai O. A Virtual Infection Model Quantifies Innate Effector Mechanisms and Candida albicans Immune Escape in Human Blood. *PLoS Comput Biol* (2014) **10**:e1003479. doi:10.1371/journal.pcbi.1003479

2. Lehnert T, Timme S, Pollmächer J, Hünniger K, Kurzai O, Figge MT. Bottom-up modeling approach for the quantitative estimation of parameters in pathogen-host interactions. *Front Microbiol* (2015) **6**: doi:10.3389/fmicb.2015.00608
